# Supplementary material for: Polo‐Like Kinase 1 Phosphorylation Tunes the Functional Viscoelastic Properties of the Centrosome Scaffold
Source: Adv Sci (Weinh). 2025 Sep 15;12(45):e11682. doi: 10.1002/advs.202511682 (PMC12677620; doi:10.1002/advs.202511682)
Supplement: Supplementary file 1 — Supporting Information [file ADVS-12-e11682-s002.docx]

**SUPPORTING INFORMATION**

**Polo-Like Kinase 1 phosphorylation tunes the functional viscoelastic properties of the centrosome scaffold**

Matthew Amato^1^, June Ho Hwang^2^, Manolo U. Rios^1^, Nicole E. Familiari^1^, Michael K. Rosen^2^, Jeffrey B. Woodruff^1,3^

1. Dept. of Cell Biology, UT Southwestern Medical Center, Dallas, TX 75390, USA
2. Dept. of Biophysics, Howard Hughes Medical Institute, UT Southwestern Medical Center, Dallas, TX 75390, USA
3. For correspondence: [jeffrey.woodruff@utsouthwestern.edu](mailto:jeffrey.woodruff@utsouthwestern.edu)

**This file contains:**

1. **Supplemental figures S1-5**
2. **Supplemental tables S1,S2**

**SUPPLEMENTAL FIGURES**

**Figure S1. In vitro analysis of PCM.**

1. Coomassie gel of proteins used in this study.
2. Reconstituted SPD-5/TPXL-1 condensates. Representative images of reconstituted SPD-5/TPXL-1 condensates prepared with 1 µM SPD-5::RFP, 1 µM TPXL-1::GFP, 50 mM KCl, 4mM Hepes pH 7.4 and incubated for 15 min.
3. SPD-5/TPXL-1 condensate fusion without kinase. Sample was prepared with 1µM SPD-5::RFP, 1µM TPXL-1::GFP, 75 mM KCl, 4 mM Hepes pH 7.4, 1 mM DTT, 0.2 mM ATP, 0.5 mM MgCl_2_) imaged after 45 min. Timelapse imaging of condensates. Arrows indicate the site of condensate fusion.
4. Representative images of reconstituted SPD-5/TPXL-1 condensates prepared with either 1 µM SPD-5:GFP(WT) or 1 µM SPD-5::GFP(4E), 1 µM TPXL-1::GFP, 50 mM KCl, Hepes pH 7.4 and incubated for 30 min.
5. Dilution assay of reconstituted PCM. 1 µM SPD-5::GFP(WT) or 1 µM SPD-5::GFP(4E), 1 µM TPXL-1::GFP was incubated in buffer (50 mM KCl, 4 mM Hepes pH 7.4, 1 mM DTT, 0.2 mM ATP, 0.5 mM MgCl_2_) with 0.5 µM PLK-1 (KD) or 0.5 µM PLK-1(CA) for 1 hr and imaged. Samples were then diluted 4.3X into buffer (250mM KCl, 25mM Hepes pH.7.4) and imaged after 1 hr (left panels). Right, quantification of SPD-5::GFP integrated density before and after dilution (mean +/- 95% C.I.; PLK-1(KD) n=6 images, PLK-1 (CA) n=6 images with >100 condensates).
6. Fluorescence recovery after photobleaching (FRAP) of reconstituted SPD-5/TPXL-1 condensates incubated with 0.5 µM PLK-1(KD) or 0.5 µM PLK-1(CA) for 45 min. TPXL-1::GFP intensity was measured and normalized to a reference condensate far from the bleach region (mean +/- 95% C.I.; KD, n = 5, CA n = 6 condensates).

**Figure S2. Effect of PP2A inhibition of PP2A on SPD-5 persistence in extruded PCM.**

1. One-cell embryo expressing wild-type *gfp::spd-5* *transgene* treated with *sur-6 RNAi* and imaged prior to pronuclear meeting*.* Look up tables adjusted for increased contrast in the right image.
2. Quantification of PCM from metaphase embryos expressing GFP::SPD-5(WT) + endogenous SPD-5 extruded into high salt buffer (150mM KCl, 25mM Hepes pH 7.4) (black curve), or high salt buffer with 10 µM LB-100 (green curve)(mean +/- 95% C.I.; no drug, n= 12; 10 µM LB-100, n=7 centrosomes).

**Figure S3. *In vivo* characterization of SPD-5(4E).**

1. PCM assembly in one-cell embryos expressing GFP::SPD-5 transgenes. Top left, representative images taken at nuclear envelope breakdown (NEBD). Bottom left, quantification of GFP::SPD-5 integrated density relative to NEBD. Top right, worms were treated with RNAi to deplete endogenous SPD-5 (mean +/- 95% C.I.; WT n=22, 4E n=22, WT + *spd-5(RNAi)* n=18, 4E + *spd-5(RNAi)* n=30).
2. Western blot against SPD-5. Alpha tubulin was detected as a loading control.
3. Time from pronuclear meeting (PNM) to nuclear envelope breakdown (NEBD) was measured in one-cell embryos expressing GFP::SPD-5 transgenes (mean +/- 95% C.I., p values from Kruskal-Wallis test followed by Dunn’s multiple comparisons test).

**Figure S4. Characterization of chromosome segregation defects in embryos expressing *gfp::spd-5 transgenes*.**

1. Quantification of chromosome segregation defects by category in Figure 6A and C (see figure 6D for n values).
2. Representative images of chromosome segregation defect categories. Unfocused spindle poles were observed in cases where centrosomes broke symmetry along the spindle axis.
3. Embryo viability assay (mean +/- 95% C.I.; WT n=8, WT + *spd-5(RNAi)* n=8, 4E n=7, 4E + *spd-5(RNAi)* n=8 mothers, >20 embryos counted per mother; p values from a Kruskal-Wallis test followed by Dunn’s multiple comparisons test).

**Figure S5. Partial FRAP of PCM in embryos expressing *gfp::spd-5* transgenes during mitosis.**

1. Top, diagram of key PLK-1 phosphorylation sites in SPD-5. Bottom, design of *gfp::spd-5* transgenes expressed at the Mos locus on chromosome II. GFP::SPD-5(2A) harbors two of the four S-to-A mutations present in GFP::SPD-5(4A).
2. Partial fluorescence recovery after photobleaching (FRAP) of PCM at nuclear envelop breakdown in one-cell embryos (no RNAi). GFP intensity was measured with a 5 μm line scan 0 s and 100 s after photobleaching (mean +/- 95% C.I.; GFP::SPD-5(WT) n=16, GFP::SPD-5(4A) n=7, GFP::SPD-5(2A) n=8 centrosomes).

**TABLE S1. Baculoviral expression constructs for protein expression.**

| **protein** | **sequence** | **plasmid name** | **parent plasmid (pOCC #)** | **N-term tag** | **C-term tag** | **species** |
| --- | --- | --- | --- | --- | --- | --- |
| SPD-5 | Full-length | JWV1 | pOCC28 | MBP-PreScission | eGFP-PreScission-6xHis | *C.elegans* |
| SPD-5 | Full-length | JWV3 | pOCC25 | MBP-PreScission | RFP-PreScission-6xHis | *C.elegans* |
| SPD-5 | Full-length | JWV10 | pOCC27 | MBP-PreScission | eGFP-PreScission-6xHis | *C.elegans* |
| TPXL-1 | Full-length | JWV29 | pOCC27 | MBP-PreScission | eGFP-PreScission-6xHis | *C.elegans* |
| TPXL-1 | Full-length | JWV30 | pOCC28 | MBP-PreScission | PreScission-6xHis | *C.elegans* |
| PLK-1(CA) | Full-length T194D | JWV11 | pOCC7 | 6xHis-Prescission | - | *C.elegans* |
| PLK-1(KD) | Full-length K67M | JWV12 | pOCC7 | 6xHis-Prescission | - | *C.elegans* |

**TABLE S2. *C. elegans* strains.**

| Strain name | genotype | Creation method | Origin |
| --- | --- | --- | --- |
| JWW12 | unc-119(ed9) III; ltSi202[pVV103/ pOD1021; Pspd-2::GFP::SPD-5 RNAiresistant;cb-unc-119(+)]II | MosSCI into EG6699 | (Woodruff et al., 2015) |
| JWW69 | unc-119(ed9) III; ltSi202[pVV103/ pOD1021; Pspd-2::GFP::SPD-5 re-encoded;cb-unc-119(+)]II; ltIs37 [(pAA64) pie-1p::mCherry::his-58 + unc-119(+)] IV. | MosSCI into EG6699 | This study |
| JWW70 | unc-119(ed9) III; utsw1[pJWB56; Pspd-2::GFP::SPD-5(530E, 627E, 653E, 658E) re-encoded; cb-unc-119(+)]II; ltIs37 [(pAA64) pie-1p::mCherry::his-58 + unc-119(+)] IV. | MosSCI into EG6699 | This study |
| JWW254 | mCherry::his-58, unc-119(ed9) III; ltSi228[pVV153/ pOD1615; Pspd-2::GFP::spd-5 S530A,S627A, S653A, S658A reencoded; cb-unc-119(+)]II | MosSCI into EG6699 | (Woodruff et al., 2015) |
| DG627 | emb-30(tn377) III. | EMS mutagenized | (Furuta et al., 2000) |
| JWW155 | emb-30(tn377) III; ltSi202[pVV103/ pOD1021; Pspd-2::GFP::SPD-5 re-encoded;cb-unc-119(+)]II; ltIs37 [(pAA64) pie-1p::mCherry::his-58 + unc-119(+)] IV. | Cross of JWW69 with DG627 | This study |
| JWW243 | emb-30(tn377) III. unc-119(ed9) III; utsw1[pJWB56; Pspd-2::GFP::SPD-5(530E, 627E, 653E, 658E) re-encoded; cb-unc-119(+)]II; ltIs37 [(pAA64) | Cross of JWW70 with DG627 | This study |
| JWW266 | emb-30(tn377) III. unc-119(ed9) III; utsw1[pJWB56; Pspd-2::GFP::SPD-5(530A, 627A, 653A, 658A) re-encoded; cb-unc-119(+)]II; ltIs37 [(pAA64) | Cross of JWW254 with DG627 | This study |
